# Extracellular Vesicle MicroRNA That Are Involved in β-Thalassemia Complications

**DOI:** 10.3390/ijms22189760

**Published:** 2021-09-09

**Authors:** Carina Levin, Ariel Koren, Annie Rebibo-Sabbah, Maya Levin, Na’ama Koifman, Benjamin Brenner, Anat Aharon

**Affiliations:** 1 Pediatric Hematology Unit, Emek Medical Center, Afula 1834111, Israel; korenariel48@gmail.com; 2 Bruce Rappaport Faculty of Medicine, Technion-Israel Institute of Technology, Haifa 3109601, Israel; b_brenner@rambam.health.gov.il (B.B.); a_aharon@yahoo.com (A.A.); 3 Department of Hematology and Bone Marrow Transplantation, Rambam Health Care Campus, Haifa 3109601, Israel; sabbah.annie@gmail.com; 4 The Hematology Research Laboratory, Tel Aviv Sourasky Medical Center, Tel Aviv 6423906, Israel; levinmaya92@gmail.com; 5 Department of Chemical Engineering and the Russell Berrie Nanotechnology Institute, Technion-Israel Institute of Technology, Haifa 3200003, Israel; movingwhat@gmail.com

**Keywords:** β-thalassemia major, extracellular vesicles (EVs), microRNA (miRNA), signal-transduction

## Abstract

Beta thalassemia major (βT) is a hereditary anemia characterized by transfusion-dependency, lifelong requirement of chelation, and organ dysfunction. MicroRNA (miRNA) can be packed into extracellular vesicles (EVs) that carry them to target cells. We explored EV-miRNA in βT and their pathophysiologic role. Circulating EVs were isolated from 35 βT-patients and 15 controls. EV miRNA was evaluated by nano-string technology and real-time quantitative polymerase chain reaction (RT-qPCR). We explored effects of EVs on cell culture proliferation, apoptosis, and signal transduction. Higher amounts of small EV (exosomes) were found in patients than in controls. The expression of 21 miRNA was > two-fold higher, and of 17 miRNA < three-fold lower in βT-EVs than control-EVs. RT-qPCR confirmed differential expression of six miRNAs in βT, particularly miR-144-3p, a regulator of erythropoiesis. Exposure of endothelial, liver Huh7, and pancreatic 1.1B4 cells to βT-EVs significantly reduced cell viability and increased cell apoptosis. βT-EV-induced endothelial cell apoptosis involved the MAPK/JNK signal-transduction pathway. In contrast, splenectomized βT-EVs induced proliferation of bone marrow mesenchymal stem cells (BM-MSC). In summary, the miR-144-3p was strongly increased; βT-EVs induced apoptosis and decreased endothelial, pancreatic, and liver cell survival while supporting BM-MSC proliferation. These mechanisms may contribute to βT organ dysfunction and complications.

## 1. Introduction

Beta thalassemia major (βT) is a severe anemia inherited in an autosomal recessive pattern. The β-globin gene maps on the short arm of chromosome 11; more than 200 disease-causing mutations have been identified. The homozygous or compound heterozygous defect causes absence or reduction in the production of β-globin chains and a consequent relative excess of α-globin. This result in ineffective erythropoiesis and progressive anemia, which requires lifetime blood transfusions and iron chelation therapy to prevent iron overload complications [1,2].

Ineffective erythropoiesis, reduction in red blood cell (RBC) lifespan, iron deposition, and free-iron toxicity are the major factors responsible for the symptomatology and complications of βT. Regular blood transfusions and iron chelation therapy markedly improve survival and quality of life [3,4,5]. However, even with proper conservative treatment, individuals with βT can develop severe complications including cardiac morbidity, liver disease, diabetes, bone disease, endocrine and cognitive dysfunctions, infections, thrombosis, and chronic pain [5,6,7]. Bone marrow transplantation is the only definitive cure but is not suitable for all patients, and gene therapy is currently under investigation in clinical trials.

βT is historically common in specific regions such as the Mediterranean area, the Middle East, and Southeast Asia. However, its prevalence has increased in the western world due to population mobility; this has created a substantial global health care problem [5]. A better understanding of the disease pathophysiology and new therapeutic approaches is required.

Extracellular vesicles (EVs) are membrane vesicles that are secreted by cells into biological fluids and that are involved in cell–cell communication. EVs include small vesicles (<100 nm), which are exosomes originating in the endosome and enriched with tetraspanin members (such as CD63 and CD81) and large vesicles (100–1000 nm), also termed microvesicles, which shed from cell surfaces [8]. We previously reported that in βT, circulating EVs reflect spleen functional status and ineffective erythropoiesis [9]. Elevated levels of platelet and RBC-EVs were found in individuals with transfusion-dependent βT/HbE who had pulmonary arterial hypertension [10]. During hypoxia, proteins that are packed in RBC EVs are involved in nitric oxide production, which leads to increased vasodilation and smooth muscle cell relaxation [11]. Exosomes were found to be involved in hepcidin regulation in the context of βT [12], and EVs were found to induce endothelial damage in sickle cell disease [13].

microRNAs (miRNAs) are small non-coding RNAs, post-transcriptional gene-expression regulators involved in several important physiological and pathological processes. These molecules are actively sorted and encased in EVs, which regulate the expression of multiple genes at a post-transcriptional level. The enrichment of miRNA in EVs is a consequence of cellular stress; several specific mechanisms are known for EV-RNA loading [14]. Circulating miRNAs have emerged as promising disease biomarkers for diagnostic and therapeutic targets in many diseases, and may be considered liquid biopsy biomarkers for disease identification, stage, and prognosis [15]. Specifically, miRNAs involved in βT were summarized in a recent review [16]. The majority of them were isolated from cells (erythroid or erythroleukemia cell lines): miR-15a/16-1, miR-486-3p, miR-26b, miR-199b-5p, miR-210, miR-34a, miR-138, miR-326, let-7, and miR-17/92 cluster elevate γ-globin expression, while miR-96, miR-146a, miR-223-3p, miR-144, and miR-451 induce α-, β-, and γ-globin expression [16]. MiR-144 inhibits γ-globin expression and is found to be involved in erythropoiesis [17,18] and apoptotic cell response [16,19,20,21]. A number of studies demonstrated dysregulation of erythroid miR-144 in sickle cell disease [22] and in β-and α-thalassemia, and the association of the disease with oxidative stress through the transcription nuclear factor erythroid 2-related factor 2 (NRF2) pathway [22,23]. Plasma exosomal miRNAs from individuals with βT were mainly positive for CD34+, indicating bone marrow origin; miR-223-3p and miR-138-5p were found to be deregulated and involved in regulating the expression of γ-globin [24].

Mitogen-activated protein kinase (MAPK) cascades have been shown to play a key role in the transduction of extracellular signals to cellular responses such as cell growth, differentiation, apoptosis, and responses to stress. At least three MAPK families have been characterized: extracellular signal-regulated kinase (ERK), c-jun N-terminal kinase (JNK)/stress-activated protein kinases (SAPK), and p38 MAPK [25,26,27]. The involvement of ERK and JNK/SAPK in EC viability/proliferation and apoptosis was previously investigated [28,29].

The current study sought to better understand the role of EVs in the multi-organ damage that characterizes β-thalassemia. Specifically, we aimed to elucidate the EV-miRNA signatures in βT, to explore the effects of EVs on cell proliferation and apoptosis in cell culture models, and to explore involved signal transduction pathways.

## 2. Results

Individuals with βT (N = 35) and healthy controls (N = 15), matched for age and gender, were enrolled to the study after signing informed consent. Blood samples were obtained in both groups; for the βT group, the samples were collected before blood transfusions and also included routine laboratory tests. The mean age of the control group was 21 ± 9 years, 7 were male, and 8 were female. The mean age of the patients was 22 ± 7 years, 18 were male, and 17 were female. Those in the βT group were genetically homozygous or compound heterozygous for the following mutations: HBB: C.93-21G>A; HBB:c.114G>A; HBB:c.118C>T; HBB:c.316-106C>G; HBB:c.78A>C; HBB:c.92C6T>C; HBB:c.92C5G>C; HBB:c.25_26delAA; and HBB:c.92C1G>A. The total βT group (N = 35) was divided into three βT subgroups based on spleen status: hypersplenism (Hy), (N = 7); no hypersplenism (no-Hy), (N = 15); and splenectomized (Sp), (N = 13). Hypersplenism was defined as cytopenia requiring 200 mL packed RBCs per kg body weight per year [9]. Those in the Sp group were slightly older; the mean age was 26 years versus 19 years in the Hy group.

For all the βT subgroups, the mean pre-transfusion Hb levels were below normal (mean 7.8 g/dL ± 0.9) and the mean ferritin level and reticulocyte count was above normal. In the Hy group, the mean white blood cell (WBC) count was below normal and the mean lactate dehydrogenase level was above normal. In the Sp group, WBC and platelet counts were higher than normal values (Table 1).

The entire βT group was transfusion-dependent. Data are expressed as mean ± SD. The normal values appear in parentheses. WBC, white blood cells; PLT, platelets; LDH, lactate dehydrogenase; no-Hy, no hypersplenism; Hy, hypersplenism; Sp, splenectomized.

### 2.1. EV Characterization

**Figure 1:**
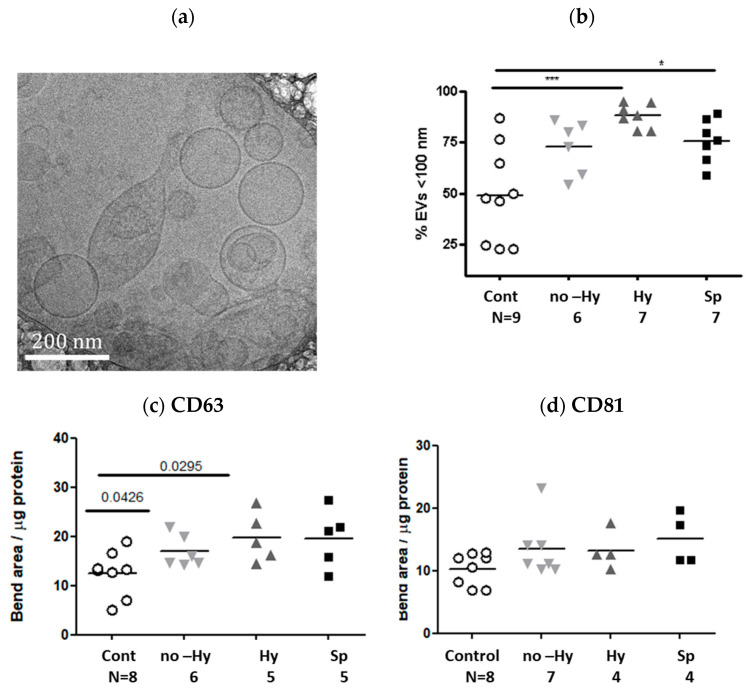
Extracellular vesicle (EV) size distribution and expression of exosome markers. (**a**) Cryogenic transmission electron microscopy, a representative image of an EV pellet from a participant in the βT group, showing a heterogeneous population of EVs. (**b**) The size distributions of EVs obtained from the healthy controls and the patient subgroups were measured by nanoparticle-tracking analysis. The graph presents the percentage of small EVs (<100 nm) in each sample. N = the number of patient samples that were validated in each subgroup. (**c**,**d**) The expression levels of EV markers (CD63 and CD81) were determined by densitometry of western blot of samples isolated from the control group and the three βT subgroups: no hypersplenism (no-Hy), hypersplenism (Hy), and splenectomized (Sp). (**c**) Expression of EV CD63. (**d**) Expression of EV CD81. * *p* < 0.05, *** *p* < 0.001.

EV size, quantity, and molecular cargo have major impacts on intracellular communication. In our work, we found important differences between the characteristics of EVs from healthy controls and from patients with βT. The electronic microscopy images displayed EVs in the range of 50–500nm (Figure 1a). Specifically, the representative Tem image showed both large EVs > 200 nm and small vesicles < 100 nm. According to the scale bar appearing in the left corner of the image, three large vesicles of >200 nm and 14 small vesicles of <100 nm were included in the current TEM image.

Nanoparticle-tracking analysis (NTA) demonstrated greater EV concentration in the total βT group (the Hy, no-Hy, and Sp subgroups together) than in the control group (0.66 E ± 2.5 EVs/μL (E + 08), (N = 9) versus 2.15 ± 1.1 EVs/μL (E + 08), (N = 20), *p* < 0.001); filtered PBS that was used for sample dilutions served as a control (6.85 ± 4.11 E + 04). The mean size of the control EVs was 80.16 ± 6.4 nm, (N = 9) and the mean size of the EVs in the total βT group was βT: 91.92 ± 22 (N = 20). The fraction of small EV (<100 nm) was larger in the βT than the control group (Figure 1b). Densitometer analysis of the gel images displayed higher expression of CD63 in the βT than the control group while expression of CD81 was found to be similar in the two groups (Figure 1c,d).

Overall, EV characterization displayed higher amounts of small EVs (exosomes) in the βT than the control group.

### 2.2. EV miRNA Profile

MiRNAs are small non-coding RNAs that are actively sorted and encased in EVs, and that regulate the expression of multiple genes at a post-transcriptional level. EV-miRNA samples from βT subgroups Sp and Hy were screened for expression levels of 800 miRNAs by nano-string technology, and compared to control samples (Supplement Figure S1).

**Figure 2:**
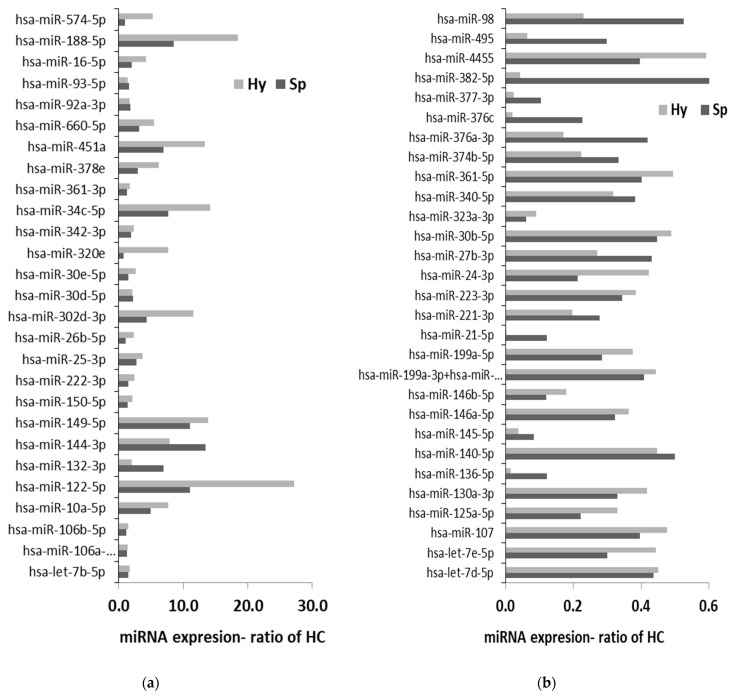
Extracellular vesicle (EV) miRNA expression screened using nano-string technology. EV pellets from two individuals each were pooled: from the hypersplenism (Hy) and splenectomized (Sp) subgroups, and from the control group. EV miRNA expression, represented as the ratio of the expression in the control group, for Hy (gray) and Sp (black). Different scales were used in (**a**,**b**) such as to emphasize the difference between two groups of miRNA. (**a**) shows miRNAs that were found to be higher in βT than control EVs, expressed as a ratio of the control group. In this graph, the scale represents the ratio > 1. (**b**) shows miRNAs that were found to be lower in βT than control EVs, expressed as a ratio of the control group. In this graph, the scale represents the ratio < 1.

The expression of 21 miRNA was > two-fold higher, and of 17 miRNA < three-fold lower in βT-EVs than control-EVs (Figure 2a). The expression of 13 miRNAs was >two-fold higher in the Sp than the Hy group, while only 8 miRNAs showed two-fold higher expression in the Hy than the Sp group (Figure 2b).

**Figure 3:**
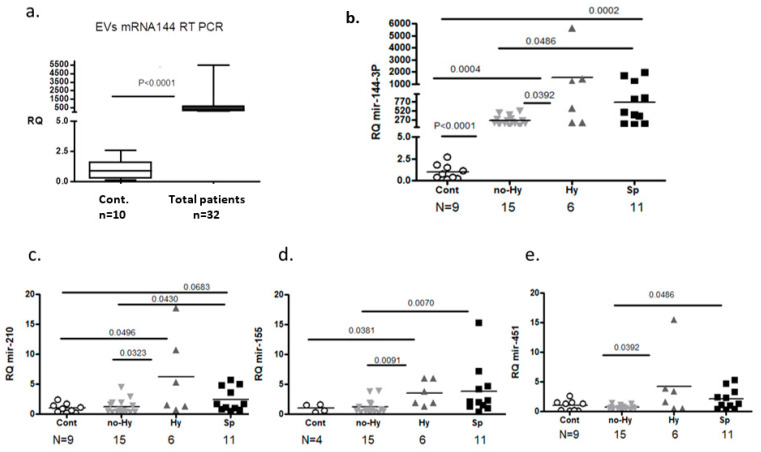
Relative expression levels of extracellular vesicle (EV) miR-144-3p by RT-qPCR. (**a**) Controls (Cont) (N = 10) vs. the total β thalassemia (Total βT) group. (**b**–**e**) Controls vs. βT subgroups (hypersplenism (Hy), no hypersplenism (no-Hy), splenectomized (Sp)) miR-144-3p, miR-201, miR-155-5p, and miR-451a, respectively. The results were normalized to cel-miR-39 spike-in expression and to the control group.

MiRNAs that presented important differences between βT samples and controls in the nano-string screening were selected for further investigation. Twenty-one distinct miRNAs were chosen for validation by real-time quantitative polymerase chain reaction (RT-qPCR). The expression of six miRNAs displayed significant differences between the βT and control groups (Table 2). Relative to the control group, the expression of miR-144-3p was higher in the total patient group (Table 2, Figure 3a) and in each of the βT subgroups (Figure 3b). Higher relative expression was found also in miR-210 (Figure 3c), miR-155-5p (Figure 3d), and miR-451a (Figure 3e) in the Hy compared with the control group (Table 2). Kyoto Encyclopedia of Genes and Genomes (KEGG) analysis pathway defined that the cluster of miR-144-3p, miR-155-5p, and hsa-miR-451a is involved in the apoptosis pathway (Supplement Figure S2).

Overall, the expression of 21 miRNA was > two-fold higher, and of 17 miRNA < three-fold lower in βT-EVs than in control-EVs. RT-qPCR confirmed differential expression of several miRNAs in βT, particularly miR-144-3p.

### 2.3. EV Effects on Cell Line Proliferation

As EVs play a major role in intercellular communication; a main goal of the current study was to evaluate the effects of EVs on essential tissues that are found to be injured in βT. Exposing cell models to EVs enabled the exploration of their effects on pathophysiological processes, and the search for specific affected cell-signaling pathways.

By using FACS AMNIS (combining flow cytometry technology and microscopic imaging), we found that EVs of the βT group penetrated to the endothelial cells (ECs) (Supplement Figure S3). Confirming internalization of EVs makes the passage of biological material possible.

**Figure 4:**
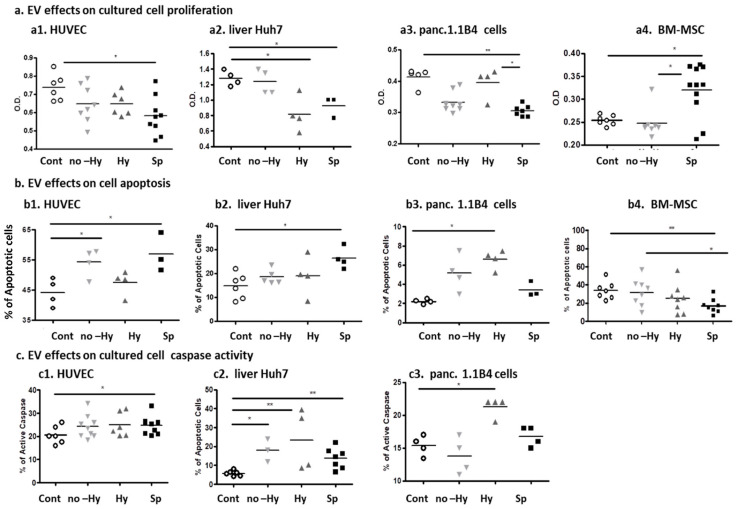
Effects of extracellular vesicles (EVs) on cell culture viability and apoptosis. Human umbilical vein endothelial cells (HUVEC), liver Huh7 cells, and pancreatic 1.1B4 and bone marrow mesenchymal stem cells (BM-MSC) were exposed to control or β thalassemia (βT) EV pellets for 20 h. (**a**) Cell viability was measured by the XTT assay (**b1–b3)** Cell apoptosis was measured by TUNEL assay; and in BM-MSC (**b4**) apoptosis was measured by Annexin/PI. (**c1–c3**) Caspase 3/7 activity assay. * *p* < 0.05, ** *p* < 0.01.

Exposure of human umbilical vein ECs (HUVECs), liver Huh7 cells, and pancreatic 1.1B4 cells to βT EV pellets for 20 h induced significant reduction in cell viability and proliferation, as measured by the XTT assay, compared with control EVs. The mean OD values and the numbers N of the samples examined were, for ECs: 0.62 ± 0.09, N = 23 vs. 0.73 ± 0.07 N = 6, *p* = 0.016; for liver Huh7 cells: 0.33 ± 0.04, n = 20 vs. 1.27 ± 0.09, N = 4, *p* = 0.014; and for pancreatic 1.1B4 cells: 0.33 ± 0.04, N = 20 vs. 0.41 ± 0.02, N = 5, *p* = 0.0048. In HUVEC and in pancreatic 1.1B4 cells, the lowest cell viability was observed in the Sp group. In liver Huh7 cells, the lowest cell viability was observed in the Hy group (Figure 4(a1–a3)). In contrast, exposure of bone marrow mesenchymal stem cells (BM-MSC) to EVs obtained from the Sp group significantly increased their proliferation (0.32 ± 0.056 OD) compared with untreated cells, and compared with treatment with control EVs (0.234 ± 0.0259, *p* = 0.0272 and 0.254 ± 0.004, *p* = 0.0298) (Figure 4(a4)).

### 2.4. EV Effects on Cultured Cell Apoptosis

HUVECs, liver Huh7 cells, and pancreatic 1.1B4 cells were exposed to βT or control EV pellets for 20 h, and apoptotic rates were validated by TUNEL assay (Figure 4(b1–b3)) and caspase 3/7 activity assay, Figure 4(c1–c3)). In both HUVECs and 1.1B4 cells, the percentage of apoptotic cells was significantly higher after exposure to EVs obtained from the total βT group than from the control group: 16.5 ± 10.3, N = 14 vs. 7.6 ± 5, N = 7, *p* = 0.01 and 5.2 ± 1.7, N = 11 vs. 2.2 ± 0.2, *p* = 0.005, respectively. The highest level was observed in the cells exposed to EVs obtained from the Hy subgroup.

In liver Huh7 cells, the percentage of apoptotic cells was slightly higher after exposure to EVs obtained from the total βT group than from the control group, 20.8 ± 6.6 N = 11 vs. 14.8 ± 5, *p* = 0.09. The highest level was observed after exposure to EVs obtained from the Sp subgroup (Figure 4(b1,b2)).

βT EVs induced higher caspase 3/7 activity in HUVECs and Huh7 cells than in EVs obtained from the control group; the percentages of positive cells were 24.5 ± 4.4, N = 24 vs. 20.5 ± 3.8, N = 6, *p* = 0.03 and 52.4 ± 6.3, N = 11 vs. 44.2 ± 4.5, N = 4, *p* = 0.43, respectively. Only exposure to Hy EVs increased the caspase activity in the pancreatic 1.1B4 cells compared with the controls (Figure 4(b3,c3)). In contrast, exposure of BM-MSC to EVs obtained from the Sp group significantly reduced their apoptosis (control 40.81 ± 21.63% vs. Hy 20.52 ± 10.45%, *p* = 0.021; or vs. Sp 16.98 ± 8.11%, *p* < 0.0019 (Figure 4(b4)). Therefore, there was no reason to validate caspase activity in these cells.

### 2.5. MAPK Involvement in EV-Induced Viability and Proliferation of ECs

**Figure 5:**
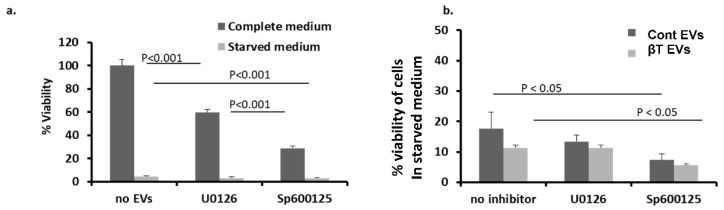
MAPK involvement in extracellular vesicle (EV)-induced viability of endothelial cells (ECs). (**a**) Human umbilical vein ECs were cultured in normal or starvation medium and incubated with or without MAPK inhibitors (U0126—MEK1/2 inhibitor and Sp600125—JNK1–3 inhibitor). (**b**) Starved ECs, cultured in medium without serum for 24 h with or without inhibitors, were stimulated with βT or control EVs (four samples for each group, in duplicates) and assessed by the XTT cell viability assay. The results are expressed as percentages of untreated (non-stimulated, non-starved) cells.

To examine MAPK involvement in EV-induced EC viability, HUVECs were cultured in complete medium or in serum-free medium (starved medium) and incubated with MAPK inhibitors U0126 (MEK1/2 inhibitor) or SP600125 (JNK1–3 inhibitor) for 1 h. In addition, serum-free medium ECs, with or without inhibitors, were stimulated with EV pellets from the βT or control group for 20 h. Four samples from each group were studied in duplicate using the XTT cell-proliferation assay. The results were expressed as the percentage of non-stimulated, non-starved (untreated) cells used as a control. Both inhibitors caused reduced viability of non-starved ECs. The reduction was higher for SP600125 than for U0126 (28.7 ± 3.1 OD vs. 59.9 ± 3.23 OD, *p* < 0.01) (Figure 5a). In serum free medium cells, the addition of EVs from healthy controls enabled partially overcoming the inhibitory effects of “starvation” conditions (15% proliferation of non-starved cells), whereas βT EVs induced only 10% proliferation.

The combination of the MEK1/2 inhibitor with EVs from either the βT or control group did not affect EC proliferation. In contrast, the combination of the JNK inhibitor with βT or control EVs significantly reduced cell proliferation compared with the starved cells exposed only to EVs (control EVs with the JNK inhibitor Sp600125: 7.33 ± 0.75 OD compared to control EVs only: 17.69 ± 6.33, *p* < 0.05; βTEVs with the JNK inhibitor Sp600125: 5.56 ± 1.95 OD compared to βT EVs only: 11.36 ± 4.114 OD, *p* = 0.057) (Figure 5b). MAPK pathways are known to be involved in a wide range of biological processes; JNK may also be involved in EV-mediated effects on cell proliferation and viability. βT EVs induced a higher apoptotic rate and caspase 3/7 activity, and reduced cell viability in ECs compared with EVs from the control group. These results suggest involvement of the MAPK signal-transduction pathway, specifically JNK, in the mechanism of apoptosis and reduced cell viability.

Overall, exposure of endothelial, liver Huh7, and pancreatic 1.1B4 cells to βT-EVs significantly reduced cell viability and increased cell apoptosis. βT-EV-induced endothelial cell apoptosis involved the MAPK/JNK signal-transduction pathway. In contrast, splenectomized βT-EVs induced proliferation of BM-MSC.

### 2.6. EV Effects on Cell hsa-miR-144-3p Expression

**Figure 6:**
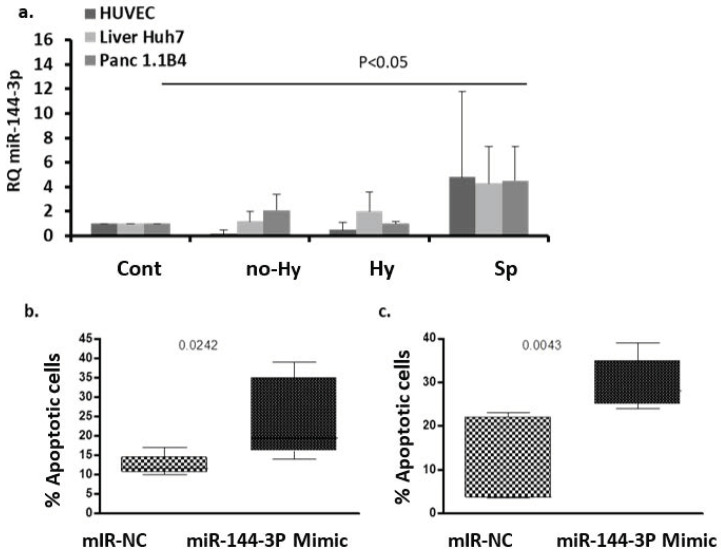
Extracellular vesicle (EV) effects on cell hsa-miR-144-3p expression. (**a**) Relative quantification of hsa-miR-144-3p in human umbilical vein endothelial cells (HUVECs), Huh7 cells, and 1.1B4 cells. The results were normalized to U6 small RNA expression and to cells after stimulation with EVs of the control group (N = 4–8). (**b**,**c**). MiR-144-3p mimic effect on cell apoptosis using the TUNEL assay. The assay was performed on liver Huh7 cells (**b**) and pancreatic 1.1B4 cells (**c**) after 24 h transfection with 30 nM miR-144-3p mimic or non-targeting negative control (miR-NC). The results are presented as the percentage of apoptotic cells of the total cell population; the data are displayed as median (horizontal bar), range from the 25th to 75th percentile (box), and extremes of distribution from 10th and 90th percentiles (error bar).

A markedly increased level of EV miR-144-3p was observed in βT, and miR-144 is known to be involved in erythropoiesis, oxidative stress, and apoptosis. To investigate an association of hsa-miR-144-3p and the effects of EV on cultured cells, we first investigated the effects of EVs of the study groups on the relative expression of cell models of miR-144-3p. No significant differences were observed in the relative expression of miR-144-3p in the total βT compared to the control group, for EV-stimulated HUVECs, Huh7 cells, or 1.1B4 cells. However, the relative expression of miR-144-3p in each of these cell models was greater in the Sp than in the control group, and than in the other βT subgroups (HUVEC 4.8 ±7, N = 9; liver Huh7 cells 2.7 ± 2.8, N = 5; pancreatic 1.1B4 cells 4.5 ± 2.8, N = 5, *p* < 0.05) (Figure 6a).

To study the effect of miR-144-3p on Huh7 and 1.1B4 cell viability and apoptosis, cells were transfected using lipofectamine for 24 h and 48 h with the specific miR-144-3p mimic mirVana™ or scramble miR (miR- negative control (NC) as a control.

#### 2.6.1. The Effect of miR-144-3p on Cell Viability

No significant difference in Huh7 or 1.1B4 cell viability was observed after transfection with miR-144-3p mimic (30 or 60 nM) compared to non-treated cells or cells treated with miR-negative control (miR-NC). Transfection efficiency was demonstrated at both concentrations by RT-qPCR.

#### 2.6.2. The Effect of miR-144-3p on Cell Apoptosis

Apoptosis was assessed in Huh7 and 1.1B4 cells after 24 h transfection with miR-144-3p.

Mimic or miR-NC by the TUNEL assay. In the Huh7 and 1.1B4 cells that were transfected with miR-144-3p, the percentage of apoptotic cells was significantly higher than in cells treated with miR-NC (23 ± 9%, N = 7 vs. 12.5 ± 3%, N = 4, *p* = 0.024 and 29.6 ± 5%, N = 5 vs. 9.7 ± 9%, N = 6, *p* = 0.0043, respectively; Figure 6b,c).

## 3. Discussion

βT represents a vast healthcare problem, including severe dysfunctions in major systems and lifelong complications. These complications can appear despite conventional treatment—periodic blood transfusions and iron-chelation therapy—thus driving the search for additional pathogenic mechanisms and novel therapies.

The current research provided specific EV-miRNA signatures in βT. These may be useful as therapeutic targets for the disease. Furthermore, the effects of EVs on cell models were evaluated and new mechanisms, such as EV-induced apoptosis, were proposed to explain the potential cell damage involved in disease complications.

The study results demonstrated small EVs of larger size in βT samples, which expressed higher levels of tetraspanins (CD63 and CD81) than control samples. This indicates a higher content of exosomes in βT than control EVs. The tetraspanin molecules are known to be involved in protein sorting from the cell membrane surface to EVs. Additionally, EV tetraspanins regulate EV targeting and uptake by recipient cells after shedding from their parental cells [30]. High expression of tetraspanins, as found in βT EVs, may characterize the EV bio-generation route and cell metabolism.

The current study defined a markedly increased EV miR-144 level in all βT subgroups. MiR-144 is known to be involved in erythropoiesis [17] and reflects erythropoietic activity [18]. Increased expression of miR-144 in RBCs was associated with severe anemia in sickle cell disease, and has been found to be involved in oxidative stress tolerance of RBCs in this disease [22]. Specifically, MiR-144 regulates NRF2 in RBCs, a regulator of cellular response to oxidative stress [23]. miR-144 appears to regulate embryonic α-hemoglobin synthesis, and this is regulated by the activity of GATA-1 [31]. In addition, miR-144 has been found to act as a tumor-suppressor gene; its inactivation or downregulation is associated with reduction in apoptosis and the progression of tumor growth via several mechanisms [19,20,21]. This supports our findings of increased apoptosis in pancreatic and hepatic cell models after transfection with miR-144 mimic. An implication of the study is that miR-144 induced apoptosis may be a contributor mechanism for diabetes, a well-known complication in thalassemia, or for hepatic dysfunction or other endocrine disorders. Future studies to support this hypothesis are required. We suggest that in βT, EV miR-144 reflects and affects these pathways, and is implicated in greater systemic dysfunction-induced apoptosis, which aggravates oxidative damage in several organs and ineffective erythropoiesis in the bone marrow.

### 3.1. EV Effects on Cell Lines

As EVs play a major role in intercellular communication; a main goal of the current study was to evaluate the effects of EVs on essential tissues that are found to be injured in βT as part of the disease pathophysiology. Exposing vascular endothelial, hepatic, and pancreatic cells to βT EVs enabled our exploring of the effects of the EVs of a number of βT subgroups on cell viability, and searching for specific affected cell-signaling pathways. Higher apoptotic rate and caspase 3/7 activity and reduced viability were observed in endothelial, liver, and pancreatic cells after stimulation with βT vs. control EVs. Previously, Kheansaard and his colleagues demonstrated that microparticles from individuals with βT/HbE induce EC dysfunction [32]. Our group previously demonstrated that monocyte-derived EVs induce apoptosis in ECs [33]. EVs from women with gestational vascular complications were shown to increase apoptosis of term trophoblast cells [34]. Another study identified the presence of caspase 3 in platelet-derived microparticles (MPs), and showed their ability to induce apoptosis in human macrophages [35]. Mechanisms of hepatic apoptosis are complicated by multiple signaling pathways [36]. The involvement of EVs in the pathogenesis of liver disease has been suggested in cirrhosis and hepatitis [37]. In the murine hepatic ischemia–reperfusion injury model, MPs circulate and can be taken up by hepatocytes, where they activate signaling pathways, including NF-κB and JNK. These mediate inflammation and hepatocyte injury [38].

In contrast to the above, Sp-EVs induced BM-MSC proliferation and reduced their apoptosis. BM-MSC are non-hematopoietic multipotent cells that are essential in hematopoiesis. They induced hematopoietic stem cell differentiation and mobilization from the BM to the circulation [39]. Under normal conditions, the niche maintains BM-MSCs in a quiescent state. However, interactions of BM–MSCs with their microenvironment affect their ability to proliferate or differentiate to other cells such as osteoblasts and adipocytes. Perturbation between stem cell proliferation and differentiation leads to stem cell depletion [40]. BM-MSCs from individuals with βT showed limited osteogenic potential, and impaired differentiation into adipocytes, due to a decrease in multipotent quiescence BM-MSCs [41]. Circulating miRNAs are involved in the onset and development of osteoporosis, a major complication observed in βT [42], and miR-144-3p inhibits the osteogenic differentiation of bone marrow-derived mesenchymal stem/stromal cells [43].

In our study, we found increased levels of miR-144-3p in βT EVs; miR-144-3p is known to induce apoptotic effects in various cells [19,20,21]. To study the effect of miR-144-3p on liver and pancreatic cell viability and apoptosis, cells were transfected with miR-144 mimic. We found greater apoptotic rates in both types of cells, showing the involvement of this miRNA in cell apoptosis.

We assume that Sp EVs play a major role in the disturbance of peripheral organs (liver, pancreas, and vasculature), while induced proliferation of BM-MSC in the BM, and the consequent suppression of osteogenic differentiation, results in osteoporosis related to βT complications.

### 3.2. The Involvement of the MAPK Pathway in EV Affects EC Viability and Apoptosis

The vascular endothelium is known to play a pivotal role in regulating blood flow and to provide potent anticoagulant properties. These prevent both initiation and propagation of the coagulation process. EC apoptosis has been implicated in numerous pathophysiological processes, such as angiogenesis, thrombosis, and atherosclerosis [44,45]. The involvement of ERK and JNK/SAPK in EC proliferation and apoptosis has been well documented in the literature [29]. Specific expression of c-Jun was found to trigger EC apoptosis [28], and JNK/SAPK is involved in the EC response to leukocyte MPs. Iron-dependent ferroptosis is a new form of cell death [46], and SP600125 is a potent ferroptosis inhibitor of autophagy and activator of apoptosis.

In our work, βT EVs induced a higher apoptotic rate and greater caspase 3/7 activity, and reduced cell viability in ECs. To examine the impact of ERK and JNK/SAPK on EV-induced EC viability, cells were treated with MEK1/2 and c-Jun inhibitors of the respective pathways. In starved cells, the addition of EVs from healthy controls partially overcame the inhibitory effects of the “stress” conditions, whereas βT EVs induced only 10% proliferation. Combining the MEK1/2 inhibitor with EVs did not affect cell proliferation. Combining the JNK inhibitor with βT or control EVs significantly reduced cell proliferation and enhanced the apoptotic effects of βT EVs. These results suggest that the JNK/SAPK signal-transduction pathway, specifically JNK, is involved in the mechanism of reduced cell viability mediated by EVs in ECs.

## 4. Methods

### 4.1. EV Isolation and Characterization

EVs were isolated as previously described [47]. In brief, blood samples were collected in tubes containing sodium citrate (3.2%) and EDTA; differential centrifugations were performed according to the current gold standard for EV isolation [48]. Specifically, platelet-poor plasma (PPP) was obtained after two sequential centrifugations (15 min 1500× *g*, 24 °C) within one hour of collection and frozen at −80 °C. EV size and concentration were validated o n PPP samples. In addition, EV pellets were isolated from thawed PPP by centrifugation (centrifuge Hettich MIKRO 220R, rotor 1189A; centrifugation condition: 1 h, 20,000× *g*, 4 °C, braking—0). The supernatant was decanted, resuspended in phosphate-buffered saline (PBS) 1:1 to the original volume for residual plasma washing, and centrifuged again. The supernatant was discarded.

According to previous reports, differential centrifugations could not provide “pure separation” between exosomes and MVs [49,50]. Storage at −80 °C was not found to have a significant effect on either EV number or size. Therefore, all the EVs were analyzed using thawed samples [51,52]. Some samples had a relatively small blood volume and therefore could not be used in all the assays. EV pellets were characterized by TEM scanning to ensure vesicle content and not cell debris. EV pellet expression of CD63 and CD81 indicated that EV samples were composed from sub-populations; those that could be considered as exosomes were tested by western blot. PPP EV size and concentration were validated by NTA. EV membrane antigens were characterized in our previous study [9] and were therefore not included in the current study.

### 4.2. EV—Morphology, Size, and Concentration

Size and concentration of EV pellets obtained from 0.5 mL PPP EVs (10 uL) were evaluated by cryogenic transmission electron microscopy and by nanoparticle-tracking analysis (NTA). (1) EV pellets were evaluated by cryogenic-TEM. Accordingly, EV pellets from one participant each from the control group and from the Hy and Sp subgroups were characterized by an FEI Talos F200C, FEG-equipped high-resolution TEM, operated at 200 kV. Specimens were transferred into a Gatan 626DH cryo-holder and equilibrated below −180 °C. Micrographs were recorded by an FEI Ceta 16 M, a 4 k × 4 k pixel, high-resolution CCD camera. Cryogenic-TEM specimens were prepared as described elsewhere [53]. Sample measurements were performed using NTA, which can measure particles in the range of 50–2000 nm [54].

PPP EVs of the control and βT groups were diluted with filtered PBS by 0.1 um membrane. Filtered PBS served as control in each measuring section. Filtered PBS and samples were analyzed with NTA version 3.1, Software Version build 3.1.54., Camera Type—sCMOS, Laser Module: NS300, 405 nm. Software settings for analysis were kept constant for all measurements. Capture Settings: Camera Level: 13; Slider Shutter: 1232; Slider Gain: 219; FPS 25.0; Number of Frames: 749; Temperature: 25 °C; Viscosity: (Water) 0.86 cP; Syringe Pump Speed: 20. At least three 30 s videos were recorded per sample in light scatter.

### 4.3. EV Pellet Exosome Markers -by Western Blot

EV-pellets were isolated from 500 µL PPP and added to buffer lysis (x2, Ray biotech) with proteinase inhibitor 1% (Sigma). A 50 µg sample in buffer lysis was combined with 2 × Laemmli sample buffer containing β-mercaptoethanol (1:20, Biorad). The samples were heated for 5 min 95 °C and separated on mini protean TGX precast gel 4–20%, and then transferred to a mini format of transfer blot turbo 0.2 µm nitrocellulose membrane (Bio-Rad, Herculs, CA, USA). Immunoblotting was performed with CD81 and CD63 (abcam). After overnight incubation with the primary antibody, the membranes were washed and incubated with horseradish peroxidase-conjugated secondary antibodies (Cell Signaling Technology, Beverly, MA, USA). Then, a chemiluminescence kit (WESTAR Nova 2, CYANAGEN, Bologna Italy) was used to detect the fluorescence. The western blot assay results were quantified using myECL™ Imager and analyzed using MyImageAnalysis Software (both from Thermo Fisher Scientific, Waltham, MA, USA).

### 4.4. Isolation of EV miRNA

MiRNA extraction was performed using miRNeasy isolation kit (Qiagen) with some modifications. Briefly, pelleted EVs obtained from 1 mL of PPP (from blood collected in EDTA tubes) were re-suspended in 200 μL double-filtered (0.2 μm) PBS. Qiazol solution (1 mL) was added and the sample was frozen at –80 °C at least overnight. The following day, after thawing, 5 fmol of cel-miR-39 spike-in (5′–3′ sequence: UCACCGGGUGUAAAUCAGCUUG, Sigma-Aldrich Israel) was added before the addition of 200 μL chloroform. After phase separation by centrifugation, glycogen (Roche Molecular Systems) was added as an RNA carrier. Isolation was performed according to the manufacturer’s instructions. The sample was eluted with 20 μL double-distilled water.

### 4.5. miRNA Profile—Nano-String Analysis

EV miRNA samples (100 ng) from each of the βT study groups Hy and Sp (pooled from two individuals each) were screened by the nano-string nCounter platform (NanoString Technologies) and compared with EV miRNA from a healthy individual. Raw data were analyzed with the nSolver software (http://www.nanostring.com/products/nSolver accessed on 31 December 2016) [55]. Heat maps of expression levels in the different samples were created with the software.

### 4.6. EV miRNA cDNA Synthesis and qPCR

Usually, a constant RNA amount (ng-µg) is used for cDNA synthesis. However, in the current study we assumed that EV RNA content may be affected by a patient’s pathophysiological condition. Therefore, RNA concentrations were validated by NanoDrop to ensure that the sample contained a sufficient amount of RNA for the kit operation, but equal volumes of mRNA were used for cDNA production. The latter was isolated from an EV pellet obtained from an equal volume of plasma (1 mL). A commercial kit (Applied Biosystems™ TaqMan™ Advanced miRNA cDNA Synthesis Kit) was used for cDNA synthesis, with 2 μL RNA as the template. The protocol was customized according to the manufacturer’s bulletin for multiplex reactions. (https://tools.thermofisher.com/content/sfs/manuals/cms_094060.pdf accessed on 28 July 2021). Pools of five specific miRNA primers were prepared (Applied Biosystems). RT-qPCR was performed in duplicate per sample using TaqMan miRNA assay and Taqman Fast Advance Master Mix (Applied Biosystems). MiRNAs from circulating EV samples were normalized to cel-miR-39 spike.

### 4.7. Cultured Cell Lines

Human primary endothelial cells (ECs). Human umbilical vein ECs (HUVECs) were used as the EC model; these were produced in the lab. Umbilical cords were obtained directly after delivery and HUVECs were isolated as previously described [34]. Cells were resuspended in culture medium, consisting of M199 with 18% (*v*/*v*) fetal calf serum (FCS), 1% (*v*/*v*) antibiotics (10,000 U/mL penicillin, 10 mg/mL streptomycin and 250 U/mL nystatin), 0.0001% (*v*/*v*) amphotericin B, 3.5 U/mL heparin, and 25 μg/mL endothelial mitogen. Cells were plated on Nunclon plates (pre-coated with fibronectin) or in flasks, incubated at 37 °C, 5% CO_2_, and used for experiments at passages 4–10.

Hepatocyte-derived carcinoma cell line (Huh7)-derived carcinoma cell line, originally from a human liver tumor HuH7 (human hepatocellular carcinoma cells). This cell line was a generous gift from Prof. Michael Aviram (The Lipid Research Laboratory, Technion Rappaport Faculty of Medicine, Haifa, Israel). Huh7, a well-differentiated hepatocyte-cell was used as the hepatic cell model. The cells were cultured in Dulbecco’s modified eagle medium (glucose 2.5% *w*/*v*) supplemented with 10% (*v*/*v*) fetal bovine serum (FBS), 100 U/mL penicillin, 100 mg/mL streptomycin, and 2 mM L-glutamine. Cells were plated on Nunclon plates (pre-coated with fibronectin) or in flasks, incubated at 37 °C, 5% CO_2_.

Pancreatic cell line: a human pancreatic β-cell line 1.1B4 was purchased from ECACC (MERCK Product Number: 10012801) and used as a human β-cell model. This cell line has demonstrated stability in culture, and enriched expression of genes encoding β cells and glucose responsiveness. The 1.1B4 cells were cultured in RPMI-1640 medium containing 11.1 mM glucose, 10% FBS, and 1% antibiotics, penicillin (100 U/mL) and streptomycin (0.1 mg/L), with 5% CO_2_ and 95% air [56].

Normal human primary bone marrow-derived mesenchymal stem cells were purchase from ATCC (BM-MSC, ATCC PCS-500-012^TM^) and were cultured in mesenchymal stem cell basal medium (ATCC) with FCS 10%, IGF-1 (15 ng/mL), and FGF-b 125 pg/mL (PEPROTEC, Israel), 1% antibiotics, penicillin (100 U/mL) and streptomycin (0.1 mg/L), with 5% CO_2_ and 95% air.

### 4.8. EV Effects on Cell Line

#### 4.8.1. Internalization of EVs by Cultured Cells

EV pellets isolated from the βT and control groups were fluorescently labeled to track their internalization into cultured cells. EVs were labeled with specific fluorescent antibodies: anti-CD41 for platelet-derived EVs and anti-glycophorin A for RBC-derived EVs. Stained EVs were added to previously detached cell lines (for example HUVECs or Huh7 cells that do not express CD41 or glycophorin A antigens) and co-incubated for 0.5–1 h at 37 °C. Finally, samples were fixed with 0.5% formaldehyde. Cells were scanned using an Amnis flow-cytometry analysis device. Cell fluorescence intensity was analyzed with Ideas 4.0, an Image Stream instrument. Cells co-incubated with unstained EVs and cells without EVs were used as negative controls [34].

#### 4.8.2. EV Effects on Cultured-Cell Viability/Proliferation and Apoptosis

Cell viability/proliferation was evaluated in ECs, Huh7 cells, 1.1B4 cells, and PBM-MSC by the XTT assay. Briefly, cells were seeded in a 96-well tissue-culture plate and cultured for 1–2 days. For each study group, EV pellets (from 250 μL PPP) were resuspended in 100 μL starvation culture medium and added to the cells, followed by 20 h incubation. The XTT assay was performed as described above, and samples were evaluated in duplicates. The results are presented as optical density (OD).

Apoptosis was evaluated in ECs, Huh7 cells and 1.1B4 cells by the TUNEL assay and caspase 3/7 activity assay. In BM-MSC, apoptosis was ensured by Annexin/PI.

Briefly, the TUNEL assay was performed according to the manufacturer’s instructions (In Situ Cell Death Detection Kit, TMR red, Roche Diagnostics). The cells were seeded in a 24-well tissue-culture plate and cultured for 1–2 days. For each study group, EVs pelleted from 2 mL PPP were re-suspended in 300 μL starvation culture medium and added to the tissue-culture plate, followed by 20 h incubation. The positive control consisted of cells treated with 50 units of DNase for 10 min. Then, cells were washed and TUNEL assay was performed according to the manufacturer’s instructions. Acquisition was performed using a Becton Dickinson CyAN FACS device. The results are expressed as the percentage of TUNEL-positive cells of the total cell population in each well.

Caspase 3/7 activity was determined with the FLICA Caspase-3/7 Kit (Immuno Chemistry Technology). Cells were seeded in a 48-well tissue-culture plate and cultured for 1–2 days. For each study group, EVs pelleted from 1 mL PPP were re-suspended in 150 μL starvation culture medium and added to the cells, followed by 20 h incubation. The positive control consisted of cells treated with 1 μM staurosporine. Cells were then detached and caspase 3/7 activity assay was performed according to the manufacturer’s instructions. Acquisition was performed using a CyAN FACS device. The results are expressed as the percentage of active caspase 3/7-positive cells of the total cell population.

#### 4.8.3. EV Involvement in Cell Signal Transductions

Phosphorylation of c-Jun (downstream of the JNK/SAPK pathway) after the addition of the JNK1-3 inhibitor (Sp600125) was evaluated in ECs by western blot analysis.

#### 4.8.4. MAPK Involvement in EV-Induced Viability/Proliferation of ECs by the XTT Assay

To examine ERK and JNK/SAPK involvement in EV-induced EC viability/proliferation, HUVECs were cultured in normal or starvation medium (medium without serum) and incubated with the inhibitors U0126 (MEK1/2 inhibitor, downstream of ERK cascade; 50 μM) and Sp600125 (JNK1-3 inhibitor; 25 μM) for 1 h. In addition, starved ECs with or without inhibitors were stimulated with EVs of the βT or control group for 20 h (four samples from each group were studied in duplicate using the XTT cell assay; the results were expressed as percentages of non-stimulated non-starved (untreated) cells used as a control).

#### 4.8.5. miRNA Extraction from Cells, cDNa Synthesis and qPCR

Cells were exposed to EVs for 6 h in reduced-serum medium. Cells were washed and total RNA was purified with TRI reagent according to the manufacturer’s instructions. The purity and concentration of the RNA was evaluated by ultraviolet absorption at 260 nm and 280 nm (NanoDrop). cDNA was constructed using 50 ng of total RNA. Pools of five specific miRNA primers were prepared (Applied Biosystems). RT-qPCR was performed in duplicates per sample using TaqMan miRNA assays and Taqman Fast Advance Master Mix (Applied Biosystems). Cell-sample miRNAs were normalized to U6 small RNA.

#### 4.8.6. miRNA Effects on Cell Lines

To study the effect of miR-144-3p on Huh7 and 1.1B4 cell viability and apoptosis, cells were transfected using lipofectamine for 24 h and 48 h with the specific miR-144-3p mimic mirVana™ or scramble miR (miR-NC) as a control, and subsequently studied using the XTT cell proliferation and TUNEL assays. Cell viability was studied after 48 and 24 h of transfection with miR-144-3p mimic or miR-NC.

Cells were seeded in a 48- or 98-well tissue-culture plate and cultured for 1–2 days. Transfection was performed when the cells were 60–80% confluent. Lipofectamine RNAiMAX (Invitrogen) was used to transfect cells with a final concentration of 30 and 60 nM miR-144-3p mimic (mirVana™) or non-targeting negative control (miR-NC) oligonucleotides (Life Technologies) according to the manufacturer’s instructions. Serum-free medium Opti-MEM^®^ was used for transfection.

Transfection efficiency of miR-144-3p (after 48 h of transfection) was assessed by RT-qPCR normalized to U6 small RNA and to cells treated, or not, with miR-NC.

#### 4.8.7. Statistical Analysis

Each experiment with each control or patient sample was performed in duplicate. The means of the duplicates were used for statistical analysis as biological replicates. Data were analyzed using GraphPad-4 software (version 4.00). Continuous variables were reported as mean ± SD. Differences between controls and patients were tested using the Mann–Whitney test. To compare subgroups, the Kruskal–Wallis test was performed, and subsequently the Dunn’s multiple comparison test. For all analyses, two-tailed test with significance *p* < 0.05 was used.

## 5. Conclusions

Overall, this study demonstrated differential miRNA expression in individuals with βT compared with healthy controls. EV-miRNA showed dysregulation of specific miRNAs involved in oxidative stress, erythropoiesis, and apoptosis. In particular, miR-144-3p was strongly increased in βT. We found that EVs of individuals with βT increased cell apoptosis in EC, hepatic, and pancreatic cell models; this mechanism might contribute to the organ damage that occurs in βT. On the other hand, these EVs increased BM-MSC proliferation, and this may disturb their ability to differentiate to osteoblasts. Moreover, we suggest the involvement of miR-144-loaded EVs as a trigger for the apoptotic process. We propose that the JNK/SAPK signal-transduction pathway, specifically JNK, is involved in the regulation of EC survival mediated by EVs. This mechanism might be implicated in the endothelial dysfunction and organ damage related to β-thalassemia. We also found several differences in EV expression between βT subgroups. However, additional studies are required to verify these findings.

In summary, EV characteristics, including membrane antigens and the content of regulatory proteins and miRNA, reflect disease severity and stages, and can serve as follow-up biomarkers of the disease dynamic. Moreover, understanding the molecular pathway may contribute to the development of new therapeutic drugs.

## Figures and Tables

**Table 1:** Blood laboratory values of the Beta thalassemia group.

Parameter, Units and Normal Values	β-Thalassemia Major Subgroups		
	no-Hy	Hy	Sp
WBC K/μL (4.5–11.)	6.9 ± 1.9	3.6 ± 1	16 ± 9
Hemoglobin g/dl (12–15)	8.7 ± 0.9	7 ± 0.9	7.8 ± 0.7
PLT K/μL (150–450)	293 ± 81	172 ± 27	919 ± 257
Reticulocytes% (0.5–2.5)	2 ± 0.9	2.1 ± 1.5	4.8 ± 3.3
Ferritin ng/mL (22–322)	1945 ± 1267	2586 ± 2000	2157 ± 1330
LDH U/L (230–480)	366 ± 93	999 ± 627	350 ± 106

**Table 2:** Relative extracellular vesicle miRNA expression by RT-qPCR.

miRNA	no-Hy (N = 10–14)	Hy (N = 6–7)	Sp (N = 13)	Total βT (N = 29–32)	*p*-Value
hsa-let-7g-5p	0.70 ± 0.5	1.77 ± 1.7	1.2 ± 0.6	1.09 ± 0.9	
hsa-miR-144-3p	221 ± 106	1494 ± 2011	713 ± 592	644 ± 1002	<0.001 †, < 0.01 #, < 0.05 ‡‡
hsa-miR-142-3p	0.71 ± 0.6	1.35 ± 1.3	1.04 ± 0.8	0.95 ± 0.8	
hsa-miR-191-5p	0.46 ± 0.4	1.38 ± 0.18	0.73 ± 0.6	0.73 ± 0.9	
hsa-miR-222-3p	0.61 ± 0.7	1.2 ± 1	1.19 ± 1.1	0.94 ± 0.94	
hsa-miR-30e-5p	0.71 ± 0.6	2.2 ± 2.4	1.68 ± 1.5	1.35 ± 1.5	
hsa-miR-20a	0.7 ± 0.7	1.87 ± 2.4	1.53 ± 1.7	1.23 ± 1.6	
hsa-miR-16-5p	0.4 ± 0.3	1.79 ± 2	1.35 ± 1.3	1.02 ± 1.3	<0.05 ‡‡
hsa-miR-195-5p	0.63 ± 0.4	2.77 ± 2.9	2.58 ± 2.8	1.76 ± 2.3	
hsa-miR-378a-3p	1.08 ± 2.6	6.3 ± 9	3 ± 5	2.8 ± 5	
hsa-miR-376	1.17 ± 1.8	2.93 ± 2.4	4.37 ± 7	2.59 ± 4	0.06 #
hsa-miR-210	1.16 ± 1.2	6.22 ± 6	2.44 ± 3.6	2.6 ± 3.6	<0.05 #,‡‡
hsa-miR-155-5p	1.14 ± 1.2	3.52 ± 2.1	3.68 ± 4	2.53 ± 2.9	<0.05 #
hsa-miR-451a	0.6 ± 0.29	4.06 ± 5.4	1.94 ± 1.6	1.75 ± 2.7	<0.05 ‡‡
hsa-miR 150-5p	1.26 ± 1	2.05 ± 1.3	1.5 ± 0.53	1.53 ± 0.9	
hsa-miR-25-3p	0.7 ± 0.43	2.6 ± 0.4	1.24 ± 1.6	1.37 ± 1.45	
hsa-miR-26b-5p	0.45 ± 0.43	1.26 ± 1.3	0.98 ± 1.1	0.79 ± 0.96	
hsa-miR-30d	0.81 ± 0.73	0.59 ± 0.43	0.29 ± 0.22	0.69 ± 0.56	
hsa-miR-150-5p	1.26 ± 1.01	2 ± 1.3	1.5 ± 0.53	1.5 ± 0.99	
hsa-miR-223-3p	0.49 ± 0.4	1.12 ± 1.7	1.24 ± 2.24	0.9 ± 1.56	
hsa-miR-342-3p	1.77 ± 2	0.86 ± 0.6	0.56 ± 0.68	1.2 ± 1.5	

Relative fold change (mean values) in miRNA expression from patient subgroups as determined by qPCR. Samples from each individual in the patient (N = 29–32) and control groups were evaluated in duplicate. The results, presented as mean values ± SD, were normalized to cel-miR-39 spike-in expression and to the control group (N = 9–11). † *p*-value between the control and total β-thalassemia (βT) groups; # *p*-value between the control and Hy groups; ‡‡ *p*-value between the βT subgroups Hy and no-Hy.

## Supplementary Materials

The following are available online at https://www.mdpi.com/article/10.3390/ijms22189760/s1.
