# Supplementary material for: Extracellular Vesicle MicroRNA That Are Involved in β-Thalassemia Complications
Source: Int J Mol Sci. 2021 Sep 9;22(18):9760. doi: 10.3390/ijms22189760 (PMC8465435; doi:10.3390/ijms22189760)
Supplement: Supplementary file 1 [file ijms-22-09760-s001.zip › ijms-1342315-supplementary.pdf]

Supplementary Materials

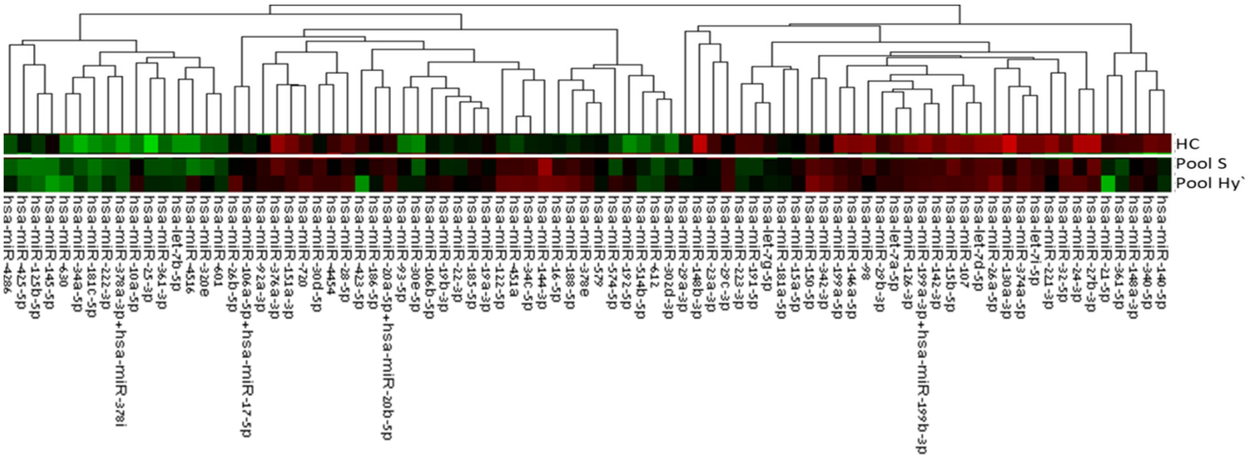

Figure S1. An miRNA heat map showing miRNA expression. This extracellular vesicle (EV) miRNA heat map shows miRNA expression in the control group and the hypersplenism (Hy) and splenectomized (Sp) subgroups. Green, black and red indicate low, intermediate and high expression, respectively. EV pellets from two individuals each, from the Hy and Sp subgroups, and from the control group were pooled.

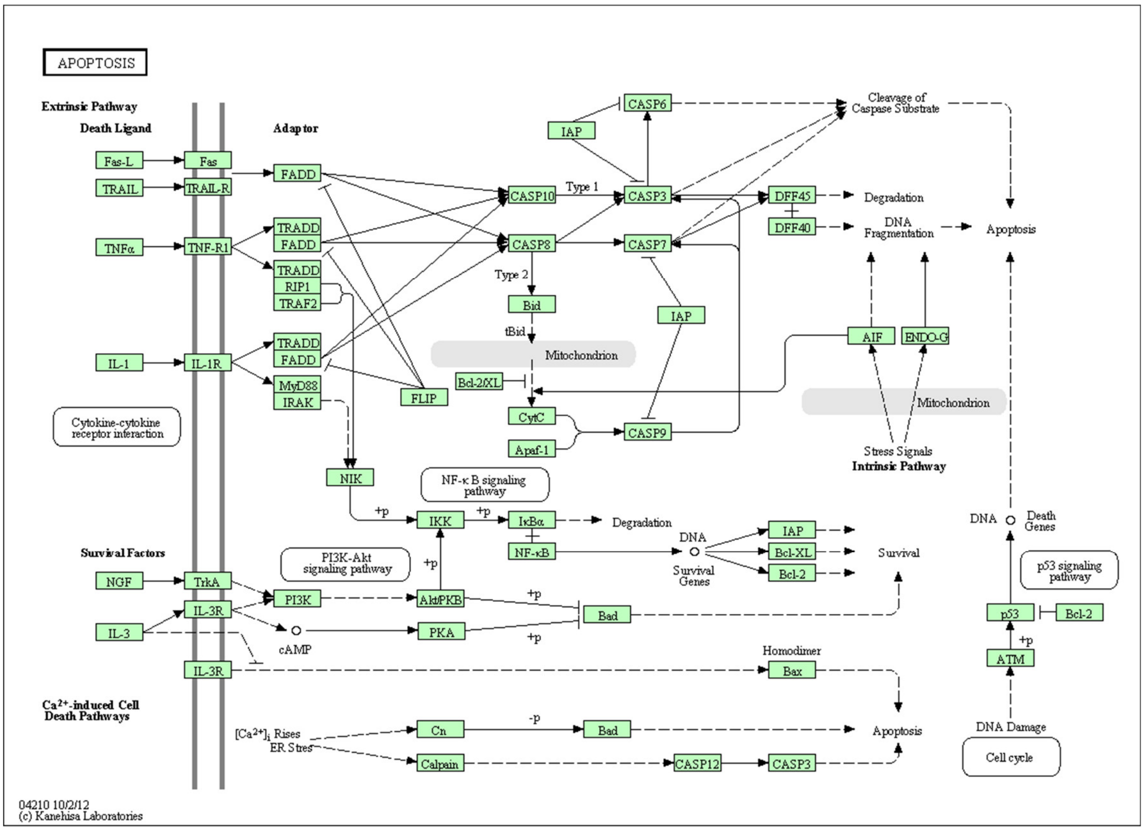

Figure S2. miRNA pathway and genes involved in apoptosis (Kyoto Encyclopedia of Genes and Genomes analysis pathway). miRs cluster: miR-144-3p, miR-155-5p and hsa-miR-451a.

Celle BF	EVs CD41-PE	Overlay

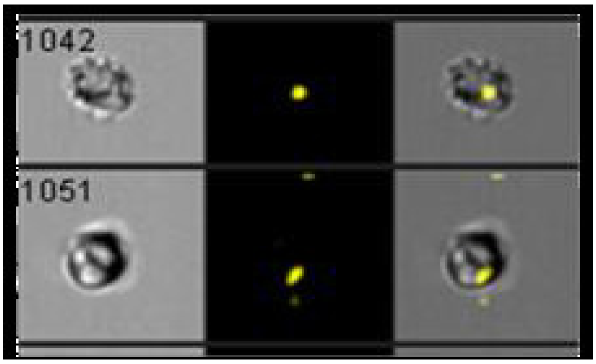

Figure S3. Extracellular vesicle cell interaction. Extracellular vesicles (EVs) labeled with anti-CD41-PE (yellow) and added to human umbilical vein endothelial cells. Images of cell uptake of the EVs were recorded in an Amnis flow cytometry device. BF, bright field (left column); fluorescence (middle and right columns).
